# Oropharyngeal squamous cell carcinomas differentially express granzyme inhibitors

**DOI:** 10.1007/s00262-016-1819-4

**Published:** 2016-03-18

**Authors:** Pauline M. W. van Kempen, Rob Noorlag, Justin E. Swartz, Niels Bovenschen, Weibel W. Braunius, Jeroen F. Vermeulen, Ellen M. Van Cann, Wilko Grolman, Stefan M. Willems

**Affiliations:** 1 Department of Otorhinolaryngology, University Medical Center Utrecht, Heidelberglaan 100, 3584 CX Utrecht, The Netherlands; 2 Department of Oral and Maxillofacial Surgery, University Medical Center Utrecht, Heidelberglaan 100, 3584 CX Utrecht, The Netherlands; 3 Department of Pathology, University Medical Center Utrecht, Heidelberglaan 100, 3584 CX Utrecht, The Netherlands; 4 Laboratory of Translational Immunology, University Medical Center Utrecht, Heidelberglaan 100, 3584 CX Utrecht, The Netherlands; 5 Department of Head and Neck Surgical Oncology, Cancer Center University Medical Center Utrecht, Heidelberglaan 100, 3584 CX Utrecht, The Netherlands

**Keywords:** Tumor microenvironment, Oropharynx squamous cell carcinoma, Human papillomavirus, Granzyme inhibitors, Tumor-infiltrating lymphocytes

## Abstract

**Objectives:**

Patients with human papillomavirus (HPV)-positive oropharyngeal squamous cell carcinomas (OPSCCs) have an improved prognosis compared to HPV-negative OPSCCs. Several theories have been proposed to explain this relatively good prognosis. One hypothesis is a difference in immune response. In this study, we compared tumor-infiltrating CD3+, CD4+, CD8+ T-cells, and granzyme inhibitors (SERPINB1, SERPINB4, and SERPINB9) between HPV-positive and HPV-negative tumors and the relation with survival.

**Methods:**

Protein expression of tumor-infiltrating lymphocytes (TILs) (CD3, CD4, and CD8) and granzyme inhibitors was analyzed in 262 OPSCCs by immunohistochemistry (IHC). Most patients (67 %) received primary radiotherapy with or without chemotherapy. Cox regression analysis was carried out to compare overall survival (OS) of patients with low and high TIL infiltration and expression of granzyme inhibitors.

**Results:**

HPV-positive OPSCCs were significantly more heavily infiltrated by TILs (*p* < 0.001) compared to HPV-negative OPSCCs. A high level of CD3+ TILs was correlated with a favorable outcome in the total cohort and in HPV-positive OPSCCs, while it reached no significance in HPV-negative OPSCCs. There was expression of all three granzyme inhibitors in OPSCCs. No differences in expression were found between HPV-positive and HPV-negative OPSCCs. Within the group of HPV-positive tumors, a high expression of SERPINB1 was associated with a significantly worse overall survival.

**Conclusion:**

HPV-positive OPSCCs with a low count of CD3+ TILs or high expression of SERPINB1 have a worse OS, comparable with HPV-negative OPSCCs. This suggests that the immune system plays an important role in the carcinogenesis of the virally induced oropharynx tumors.

## Introduction

The causative role of the human papillomavirus (HPV), especially in oropharynx squamous cell carcinoma (OPSCC), is widely established [1]. Studies showed prevalence rates varying between 15 and 90 % of HPV-positive tumors in OPSCC worldwide and an increase is reported in the last two decades [2, 3]. Despite their general presentation at a more advanced stage, HPV-positive OPSCCs have a better survival compared to HPV-negative OPSCCs [4, 5].

The exact cause of this survival advantage is not fully elucidated and seems to be related to multiple factors such as the absence of field cancerization and wild-type expression of p53 and Rb [6]. Additionally, non-oropharyngeal HPV-positive head and neck squamous cell carcinoma (HNSCC) does not show this improved outcome compared to their HPV-negative counterparts [7]. This observation suggests that the behavior of a HNSCC induced by HPV might be influenced by the tissue microenvironment of that specific subsite. The subepithelial tissue in the oropharynx is mainly lymphoid, indicating that especially the immune system might harbor an important clue in the different behavior of HPV-positive tumors in the oropharynx.

For many cancers, it has become clear that in addition to genetic and epigenetic changes in the neoplastic cells, also microenvironmental factors play an important role in carcinogenesis and tumor progression. Cancers can survive the host immune response by escaping recognition and suppression of the host immune system [8]. Indeed, several studies show an increased immune response in HPV-positive OPSCC in the primary tumor which may contribute to their improved treatment response [9–11].

Recognition of the important regulatory role of the immune system on the behavior of cancer cells has led to the awareness that manipulating the microenvironment could be used as therapeutic target. Indeed, stimulating the immune response against the cancer cells has been shown very successful in various cancer types [12, 13]. However, effective immunotherapy can only be achieved when potential mechanisms of the tumor to escape from recognition or killing by the immune system are overcome. Moreover, expression of intracellular serine protease inhibitors (SERPINS) by tumors could help the tumor cells to escape from granzyme-mediated apoptosis pathways and can lead to a poor response to cellular immunotherapy [8]. This pathway is the major mechanism via which cytotoxic lymphocytes eliminate virus-infected and tumor cells by releasing granules containing serine proteases (granzymes) to induce apoptosis [14, 15]. Humans express five different granzymes ((Gr) GrA, GrB, GrK, GrH, and GrM)) and intracellular inhibitors for GrB, GrM, and GrH have been identified, being SERPINB9, SERPINB4, and SERPINB1, respectively [16–19].

We investigated the intratumoral immune cell response, as well as the expression of these intracellular granzyme inhibitors in OPSCC as a potential mechanism for tumor cell immune evasion and correlated their expression with clinicopathological variables such as HPV status and overall survival.

## Materials and methods

### Patient selection and clinicopathological information

Our study cohort involved 288 patients with histologically proven primary OPSCC curatively treated at the University Medical Center Utrecht, the Netherlands, between 1997 and 2011 of which formalin fixed paraffin-embedded (FFPE) tissue blocks of the primary tumor were available in our pathology archives. The tissue blocks were incisional biopsies, as the treatment was mostly primary radiotherapy or chemoradiotherapy.

Since the tissue used was a remainder following the clinical diagnostic process, no ethical approval was required according to Dutch national ethical guidelines (www.federa.org). Anonymous or coded use of leftover tissue for scientific purposes is part of standard treatment agreement with patients in our center [20].

Tissue microarrays were constructed as described previously [21]. Briefly, hematoxylin–eosin-stained slides were examined and revised by a dedicated head and neck pathologist (S. M. Willems) to mark representative tumor regions. Four tissue microarrays (TMA) were constructed using a fully automated tissue microarray instrument (TMA Grand Master, Beecher Instruments). From each tumor block, three cores with a diameter of 0.6 mm were punched out and placed in a recipient paraffin block. Areas including necrosis were avoided. In addition, normal oropharynx mucosa obtained from patients with neck metastasis from an unknown primary tumor was incorporated in each block as a control among the different TMAs. Nowadays, TMAs are accepted as a fast and accurate approach for the evaluation of immunohistochemical stainings in large groups [22].

From all included OPSCCs, patient characteristics, clinical TNM classification (based on palpation, ultrasound-guided fine-needle aspiration, MRI or CT, and classified in a multidisciplinary panel) based on the AJCC TNM classification of malignant tumors 7th edition and survival data were extracted from medical records. HPV status was determined for all tumors by a combination of p16 immunohistochemistry and linear array, as described earlier [21]. Adequate DNA for HPV testing and enough percentage tumor cells (>30 %) for constructing a TMA was available for 262 cases of the initial 288 OPSCC cohort (91 %).

### Immunohistochemistry

Immunohistochemical evaluation of tumor-infiltrating lymphocytes, GrB, and the three granzyme inhibitors (SERPINB1, SERPINB4, and SERPINB9) was performed on 4 µm paraffin TMA slides. Sections were heated at 60 °C overnight. All tissue sections were deparaffinized with xylene and rehydrated in decreasing ethanol dilutions. Endogenous peroxidase activity was blocked for 15 min in a 0.3 % hydrogen peroxidase phosphate-citrate buffer (pH 5.8) followed by antigen retrieval. For CD3+, CD4+, CD8+, granzyme B, and SERPINB1, IHC staining was performed using an automated immunostainer (Benchmark Ultra, Ventana, Roche). All other stainings were performed manually. After antigen retrieval by boiling the sections in EDTA buffer pH 9.0 or citrate buffer pH 6.0 for 20 min, sections were cooled within the buffers for 30 min followed by incubation with the primary antibodies for 60 min. The used antibodies, antigen retrieval, and appropriate dilutions are summarized in Table 1. For the detection of the primary antibodies, a poly-HRP anti-mouse, rabbit, rat (ready to use: Brightvision, DPVO-HRP, immunologic), or in case of SERPINB4, the Novolink kit (Leica) was used according to manufacturer’s protocol. Between steps, slides were washed three times with PBS. Slides were then developed with diaminobenzidine for 10 min followed by counterstaining with hematoxylin, dehydration in alcohol and mounting. Finally, the sections were coverslipped. Appropriate negative and positive controls were used in all stainings.

**Table 1:** Overview of used antibodies

	Antigen retrieval	Clone	Company	Dilution	Positive control
P16	EDTA	16P07	Neomarkers	1:200	Tonsil
CD3+	EDTA	A452	Dako	1:100	Tonsil
CD4+	EDTA	SP35	CellMarque	1:50	Tonsil
CD8+	Citrate	M7103	Dako	1:50	Tonsil
SERPINB1	EDTA	Ab47731	Abcam	1:800	Pancreas tumor
SERPINB4	Citrate	10C12	Santa cruz	1:50	Skin
SERPINB9	Citrate	Clone 17	Ref. [44]	1:400	Tonsil
Granzyme B	Citrate	GB7	Ref. [45]	1:250	Tonsil

### Evaluation of immunohistochemical staining

Scoring of TMA cores was performed mutually by a dedicated head and neck pathologist (S. M. Willems) and a researcher (P. M. W. van Kempen), both blinded to clinical characteristics of the patients. A core was excluded when >95 % of the core contained no tissue or when the core contained <5 % tumor tissue. Discordance was resolved by discussion and consonance. Patients were only included in the analyses if one or more tumor cores were available. For CD3+, CD4+, and CD8+, the number of positively stained lymphocytes infiltrating the tumor tissue was counted. For SERPINB1 and SERPINB4, the percentages of nuclear or cytoplasmic stained tumor cells were scored. For SERPINB4, staining intensity was scored as well and evaluated as absent (0), weak (1), moderate (2), and strong (4). SERPINB9 was scored as positive (staining in more than 10 % of tumor cells) or negative as used in previous studies [23]. We investigated the expression of SERPINS in neoplastic epithelial cells, although SERPINS are also expressed in other cell types, e.g., lymphocytes and fibroblasts where they exhibit specific functions [24].

When more than one core was available for one case, the mean number (CD3+, CD4+, CD8+) or the mean percentage (SERPINB1, SERPINB4) was calculated. For SERPINB4, a *H*-score was calculated by multiplying the mean percentage of SERPINB4 staining with the maximum staining intensity. For all these proteins, an optimized cutoff point with regard to overall survival was identified using ROC curves. For CD3+, the optimal cutoff value was 150, and for CD4+ and CD8+, it was 100. For SERPINB1 and SERPINB4, it both was 50 %, and using the *H*-score for SERPINB4, the threshold was identified at 150. The activity of cytotoxic tumor-infiltrating lymphocytes was examined by GrB expression. Per tumor core, the number of GrB-positive cells were counted and divided by the number of cytotoxic lymphocytes.

#### Statistical analyses

SPSS IBM, version 22.0, was used for all analyses. Differences in patient characteristics between HPV-positive and HPV-negative OPSCCs were calculated using the Pearson’s chi-squared test for categorical variables and the student’s *t* test for normally distributed continuous variables. The Mann–Whitney U test was used to assess differences in the expression of CD3+, CD4+, CD8+, SERPINB1, and SERPINB4 between the HPV-positive and HPV-negative OPSCCs as these continuous variables were non-normally distributed. Correlations were analyzed using Pearson correlation. Overall survival (OS) was used as outcome endpoint and defined as the time between date of first tumor positive biopsy and death. Censored patients were confirmed alive at time of censoring. Thus, there was no loss to follow-up. Survival rates were plotted according to the Kaplan–Meier method, and associations were analyzed using the log-rank test. Effect modification and confounding were investigated. Multivariate analyses were performed using the Cox proportional hazard model in a stepwise-backward selection procedure including the variables that were significant in univariate analysis. Receiver operating characteristic (ROC) curves analyses were used to determine cutoff points for protein expression and survival. Statistical significance was set at *p* < 0.05.

## Results

### Patients’ characteristics

The study included 262 OPSCC patients with a mean age of 59 at primary diagnosis. Overall, 17 % of the OPSCCs were HPV-positive and 83 % were HPV-negative. Patient and tumor characteristics are depicted in Table 2. In line with the literature, patients with HPV-negative tumors had higher T-stages compared to patients with HPV-positive tumors, but there were fewer cases of nodal metastases. With regard to smoking and alcohol abuse, patients with HPV-positive tumors used significant less alcohol. There was no significant difference between the groups considering smoking. Cisplatinum-based chemoradiotherapy (CCRT) was the main treatment of patients (Table 2). CCRT was administered to patients below 70 years of age, if tumors were functionally or technically inoperable (T3–T4 tumors) and if there were no further contra-indications to undergo this treatment.

**Table 2:** Patient and tumor characteristics of 262 OPSCC by HPV status

Patient or tumor characteristics	HPV-positive (%)	HPV-negative (%)	*p* value
No. of cases	44 (17)	218 (83)	–
Age			
Average (range)	58 (35–80)	59 (39–83)	0.225
Sex			
Male	34 (77)	148 (68)	
Female	10 (23)	70 (32)	0.292
Smoking history^a^			
Never or quit >1 year	25 (57)	94 (43)	
Yes or quit <1 year	19 (43)	122 (56)	0.148
Alcohol use^b^			
Never or quit >1 year	26 (59)	60 (28)	
Yes or quit <1 year	18 (41)	154 (71)	<0.001
Overall AJCC stage^c^			
Stage I-II	5 (11)	42 (19)	
Stage III-IV	39 (89)	175 (80)	0.297
AJCC tumor size^c^			
T1–2	24 (55)	85 (39)	
T3–4	19 (43)	133 (61)	0.041
AJCC nodal stage^d^			
N0	5 (11)	86 (39)	
N1-3	39 (89)	130 (60)	<0.001
Treatment			
Surgery only	1 (2)	14 (6)	
Surgery + PORT	9 (20)	57 (26)	
Surgery + PORCT	1 (2)	4 (2)	
Primary RT	13 (30)	56 (26)	
Primary CCRT	20 (46)	87 (40)	0.738
Second primary tumors^a^			
No	43 (98)	199 (91)	
Yes	1 (2)	17 (8)	0.314
Intratumor CD3+ cells			
0–150	18 (42)	186 (87)	
>150	25 (58)	28 (13)	<0.001
Intratumor CD4+ cells			
0–100	21 (49)	172 (81)	
>100	22 (51)	41 (19)	<0.001
Intratumor CD8+ cells			
0–100	16 (41)	177 (85)	
>100	23 (59)	31 (15)	<0.001
SERPINB9 expression			
0–10 %	36 (97)	189 (95)	
>10 %	1 (3)	9 (5)	0.947
SERPINB1 expression			
0–49 %	30 (75)	141 (70)	
>50 %	10 (25)	61 (30)	0.639
SERPINB4 expression			
0–49 %	19 (49)	80 (39)	
>50 %	20 (51)	124 (61)	0.353
SERPINB4 intensity			
Absent/weak	18 (46)	84 (41)	
Moderate/strong	21 (54)	120 (59)	0.689

*HPV* Human papillomavirus, *PORT* postoperative radiotherapy, *RT* Radiotherapy, *CCRT* cisplatinum based chemoradiotherapy, *AJCC* American Joint Committee on Cancer. Due to insufficient tissue a few cases could not be evaluated for CD3+ (5), CD4+ (6), CD8+ (15), SERPINB1 (20), SERPINB4 (19) and SERPINB9 (27) staining

^a^ 2 missing; ^b^ 4 missing values; ^c^ 1 missing; ^d^ 2 missing

### HPV-status and tumor infiltration by immune cells

**Fig. 1:**
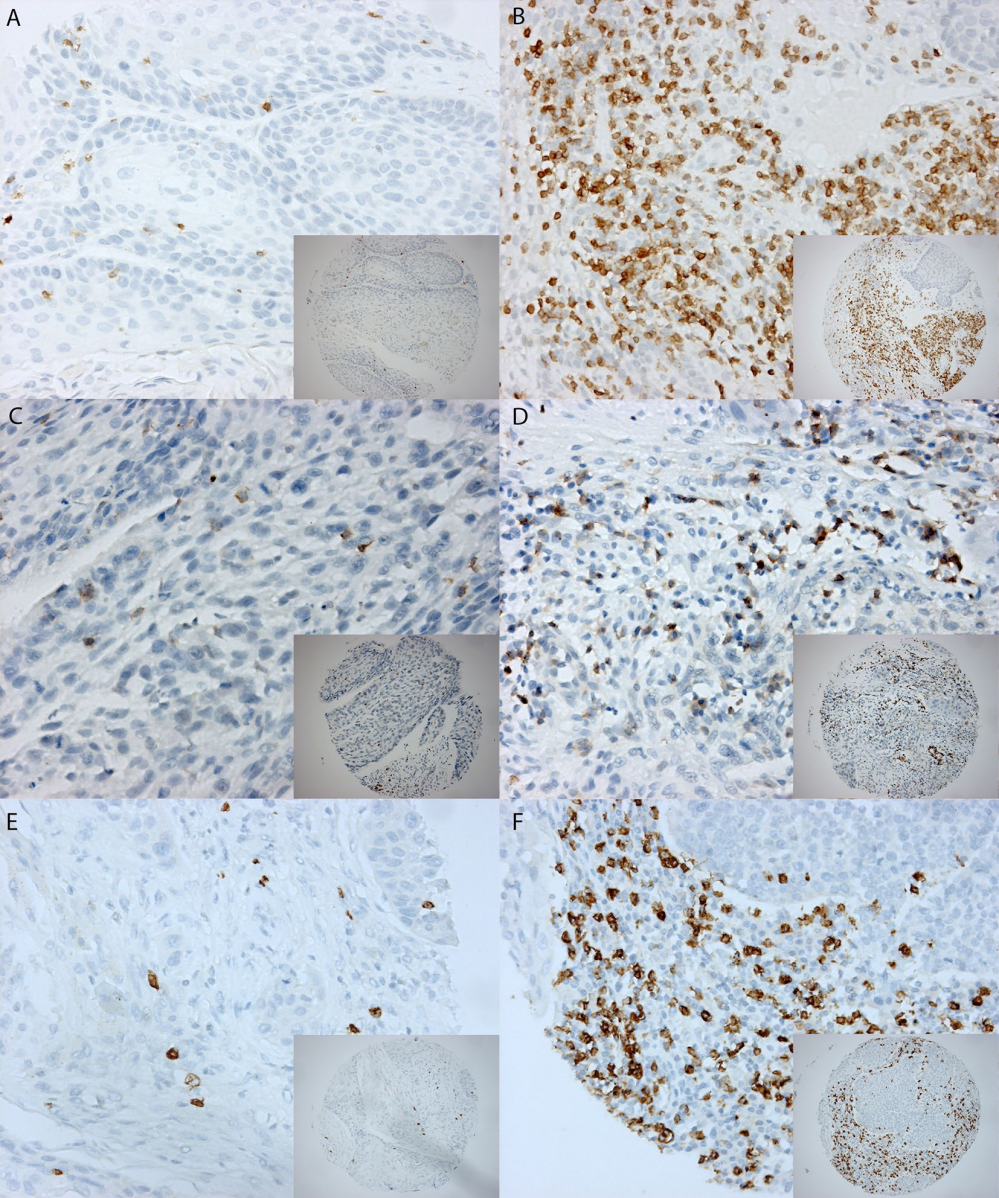

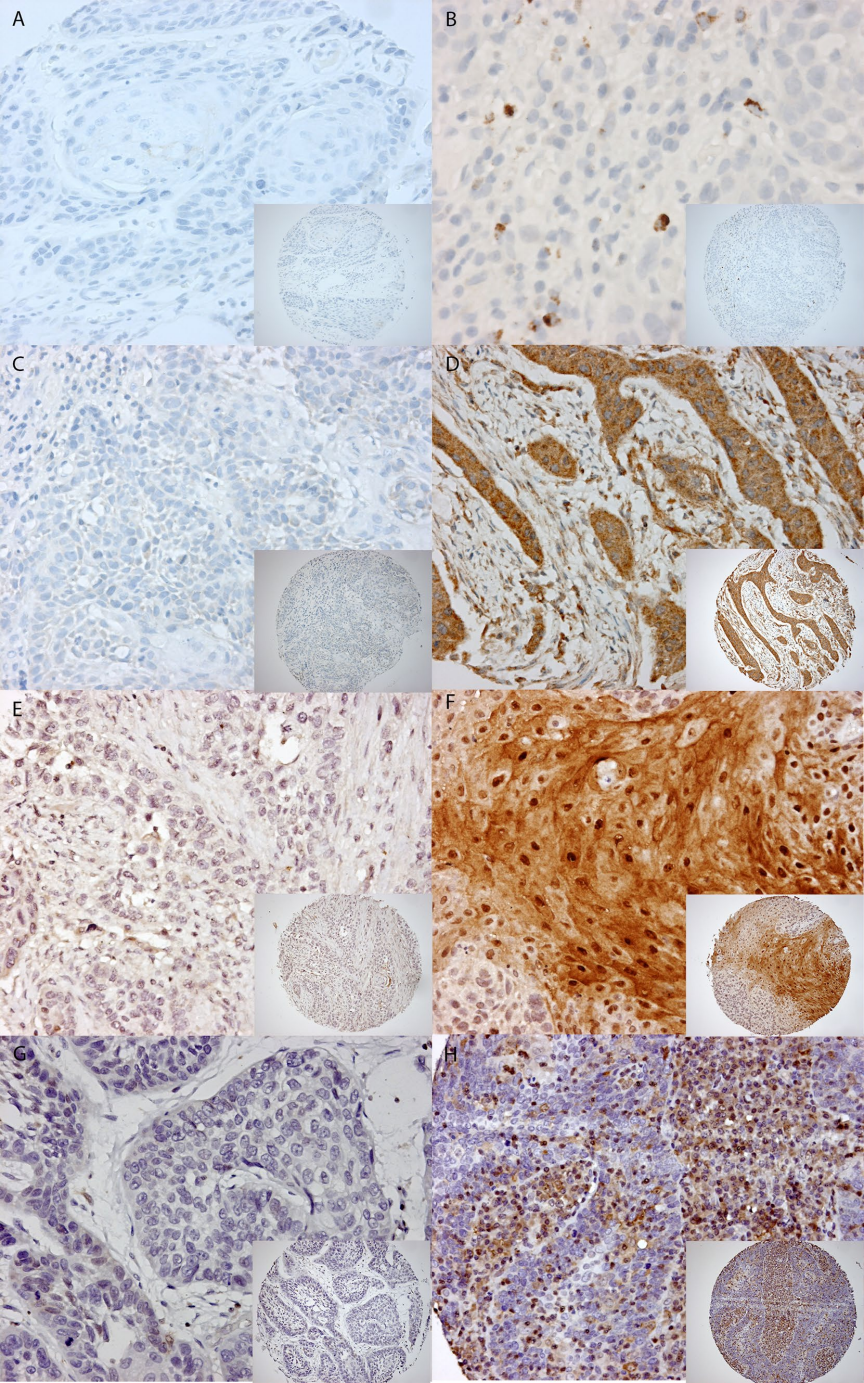
**a** Staining patterns of CD3+, CD4+, and CD8+. Example of a 0.6 mm TMA core with low expression on the *left side* (**A**) and high expression on the *right side* for CD3+ (**B**) (magnification 10× and magnification 20×). The same for CD4+ (**C**, **D**) and CD8+ (**E**, **F**). **b** Staining patterns of granzyme B, SERPINB1, SERPINB4, and SERPINB9. Example of a 0.6 mm TMA core with low expression on the *left side* compared to high expression on the *right side* for granzyme B (**A**, **B**), SERPINB1 (**C**, **D**), SERPINB4 (**E**, **F**), and SERPINB9 (**G**, **H**) (magnification 10× and magnification 20×)

Due to insufficient tissue, a few cases could not be evaluated for CD3+, CD4+, or CD8+ staining (Table 2). The number of intratumoral CD3+, CD4+, and CD8+ cells was assessed and compared between HPV-positive and HPV-negative OPSCCs (Fig. 1a). The mean number of intratumor CD3+, CD4+, and CD8+ cells was significantly higher in HPV-positive tumors. The number of tumor-infiltrating CD3+, CD4+, and CD8+ positive cells was dichotomized (CD3+ with a cutoff value of 150; CD8+ and CD4+ with a cutoff value of 100) and compared between HPV-positive and HPV-negative tumors. A high number (>150) of tumor-infiltrating CD3+ cells was observed in 28 of 214 (13 %) HPV-negative tumors compared to 25 of 43 (58 %) HPV-positive tumors (*p* < 0.001). Furthermore, a high number intratumor CD4+ and CD8+ positive cells (>100) was found in 41 of 213 (19 %) and 31 of 208 (15 %) HPV-negative tumors versus 21 of 43 (49 %) and 23 of 39 (59 %) HPV-positive tumors. These differences were significant (*p* < 0.001).

### Expression of granzymes inhibitors

In normal tissue of the oropharyngeal region SERPINB9, SERPINB1, and SERPINB4, expression was absent. For SERPINB9, SERPINB1, and SERPINB4, we mainly observed a cytoplasmic staining with sometimes nuclear staining as well (Fig. 1b). The proportion of patients with positive and negative SERPIN staining is depicted in Table 2. Even though SERPIN expression was in all cases lower in HPV-positive tumors than in HPV-negative tumors, none of these differences reached statistical significance. SERPINB9 expression could be scored in 235 OPSCCs (90 %). The expression level of SERPINB9 was heterogeneous and positive in 28 (12 %) of 235 evaluable cases. There was no significant difference in SERPINB9 expression between HPV-positive and HPV-negative tumors. SERPINB1 and SERPINB4 expression could be scored in 242 (92 %) and 243 (93 %) of the cases, respectively. Using a cutoff value of 50 %, 71 tumors of the 242 (29 %) showed a high expression of SERPBINB1 in the total cohort. The HPV-positive OPSCC showed a high expression of SERPINB1 in 25 % (10 of 40) compared to 30 % (61 of 202) of HPV-negative, though this difference was not significant (*p* = 0.639). SERPINB4 staining intensity, as well as the percentage of SERPINB4 positive tumor cells, was compared between HPV-positive and HPV-negative OPSCC. The SERPINB4 intensity, dichotomized as absent (0)/weak (1) versus moderate (2)/strong (3), was observed as high (moderate/strong) in 18 of 39 (46 %) of HPV-positive OPSCC compared to 120 of 204 (59 %) HPV-negative tumors (*p* = 0.564). Using a cutoff value of 50 %, 20 of 39 (51 %) HPV-positive compared to 124 of 204 (61 %) HPV-negative tumors showed a high SERPINB4 expression (*p* = 0.353). Results are summarized in Table 2.

We correlated expression of intratumoral CD3+, CD4+, and CD8+ to expression of the granzyme inhibitors. There was no significant correlation between expression of TIL’s and granzyme inhibitors. GrB expression was evaluated to determine the activity of cytotoxic tumor-infiltrating lymphocytes. Tumors that showed SERPINB9 expression (>10 %) contained a significant higher percentage of granzyme B-positive cytotoxic T lymphocytes (CTLs) (mean granzyme B-positive CTLs 23 vs. 38 % in SERPINB9 positive tumors).

### Effect of tumor infiltration by immune cells and granzyme inhibitors on survival

Univariate analyses using Kaplan–Meier curves were performed to evaluate factors potentially associated with OS. The analyses were performed in the total cohort, as well as stratified for patients with HPV-positive and HPV-negative tumors The median follow-up was 35 months for all patients (interquartile range [IQR]: 15.8–67.0). Of the total cohort of 274 patients, 151 (55 %) patients had died at the end of follow-up.

**Fig. 2:**
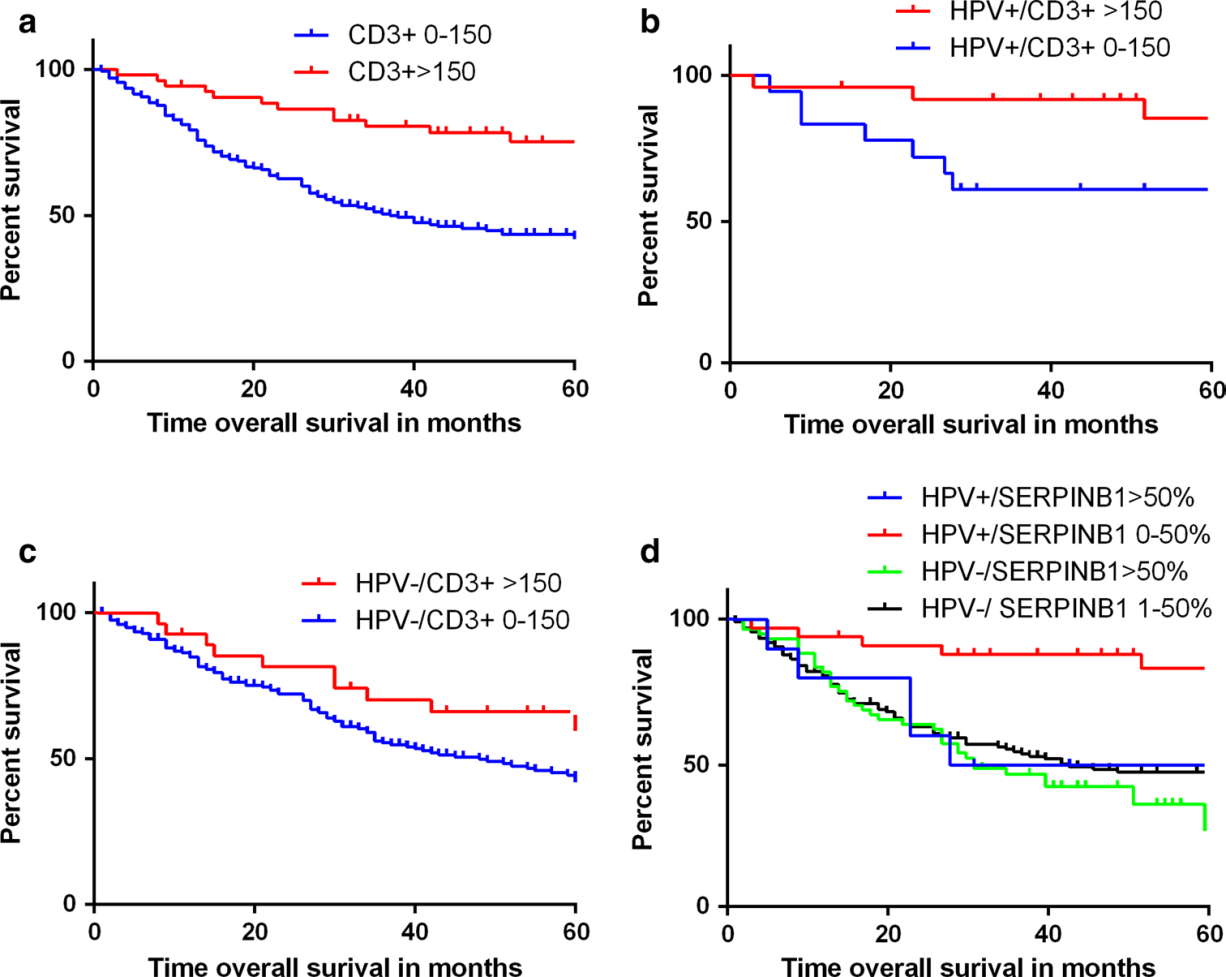
Kaplan–Meier curves for OPSCC overall survival. Kaplan–Meier curves of OS. **a** For high levels (>150) of intratumoral CD3+ cells versus low levels in the total cohort of OPSCC. Log-rank *p* < 0.001, hazard ratio = 0.36 (0.21–0.61), *p* < 0.001. **b** For HPV-positive tumors. Log-rank *p* = 0.029, hazard ratio = 0.29 (0.09–0.95), *p* = 0.042 **c** For HPV-negative tumors. Log-rank *p* = 0.060, hazard ratio = 0.61 (0.37–1.03), *p* = 0.065. **d** Kaplan–Meier OS curve for SERPINB1 expression stratified for HPV status. It shows that the OS of HPV-positive tumors with a high SERPINB1 expression is comparable to HPV-negative patients

Prognostic factors included in the univariate analysis were as follows: age, gender, tumor size (cT1-2 vs. cT3-4), smoking, alcohol use, nodal stage (cN0 vs. cN+), number of intratumor CD3+, CD8+, and CD4+ cells, SERPINB9 staining (positive vs. negative), SERPINB1 staining (high vs. low expression), and SERPINB4 staining (H-score high vs. low expression). In the total group, HPV-positivity, small tumor size (T1–T2), no lymph node metastasis, high number of CD3+ and CD8+ intratumor cells, and a low expression of SERPINB1 in tumor cells were all individually significant associated with an improved OS (Table 3, Fig. 2). The Cox proportional hazard regression model showed that tumor size (T1–T2), nodal stage (N0), HPV positivity, and a high number of intratumor CD3+ cells were independent prognostic factors for an improved OS (Table 4).

**Table 3:** Univariate models for overall survival

Overall survival	Total cohort		HPV-positive OPSCC		HPV-negative OPSCC	
	Hazard ratio (95 % CI)	*p* value	Hazard ratio (95 % CI)	*p* value	Hazard ratio (95 % CI)	*p* value
Gender (male vs. female)	0.85 (0.59–1.23)	0.387	0.34 (0.04–2.68)	0.305	0.86 (0.59–1.26)	0.434
Age (>60 vs. younger)	1.08 (0.78–1.51)	0.644	3.97 (1.02–15.4)	**0.046**	0.93 (0.66–1.31)	0.670
Tumor size (T3–4 vs. T1–2)	2.10 (1.45–3.00)	**<0.001**	3.19 (0.82–12.4)	0.093	1.90 (1.32–2.74)	**<0.001**
Nodal stage (N+ vs. N0)	1.82 (1.26–2.62)	**<0.001**	1.14 (0.14–9.00)	0.902	2.55 (1.73–3.77)	**<0.001**
Smoking (current/quit vs. never ever)	0.78 (0.56–1.09)	0.148	0.77 (0.53–1.11)	0.159	0.79 (0.56–1.11)	0.176
Alcohol use (current/quit vs. never ever)	1.00 (0.71–1.43)	0.97	1.53 (0.44–5.29)	0.501	0.77 (0.53–1.11)	0.768
Number of CD3+ cells (>150 vs. 0–149)	0.36 (0.21–0.61)	**<0.001**	0.29 (0.09–0.95)	**0.042**	0.61 (0.37–1.03)	0.065
Number of CD4+ cells (>100 vs. 0–99)	0.66 (0.43–1.00)	0.051	0.58 (0.16–2.05)	0.393	0.84 (0.54–1.32)	0.456
Number of CD8+ cells (>100 vs. 0–99)	0.37 (0.22–0.62)	**<0.001**	0.31 (0.08–1.24)	0.100	0.57 (0.32–1.01)	0.054
SERPINB1 (>50 % vs. 0–49 %)	1.60 (1.11–2.31)	**0.012**	4.58 (1.22–17.1)	**0.024**	1.37 (0.93–2.00)	0.110
SERPINB4 (H-score) (>150 vs. 0–149)	1.12 (0.79–1.58)	0.527	1.92 (0.56–6.63)	0.304	1.05 (0.73–1.50)	0.812
SERPINB9 (>10 % vs. 0–9 %)	0.84 (0.47–1.50)	0.563	0.05 (0.00–7397)	0.637	0.75 (0.42–1.34)	0.330
HPV (positive vs. negative)	0.28 (0.15–0.54)	**<0.001**	–	–	–	–
Treatment (CCRT vs. surgery)	1.41 (0.98–2.01)	**0.062**	34.67 (0.11–11372)	0.230	1.38 (0.96–1.99)	0.081

Significant values are shown in bold

*HPV* Human papillomavirus, *OPSCC* oropharyngeal squamous cell carcinoma, *CI* confidence interval, *CCRT* radiotherapy with or without adjuvant chemotherapy

**Table 4:** Multivariate model for overall survival in total cohort and for HPV-negative OPSCC

Overall survival	Total cohort OPSCC		HPV-negative OPSCC	
	Hazard ratio (95 % CI)	*p* value	Hazard ratio (95 % CI)	*p* value
Tumor size (T3-4 vs. T1-2)	1.73 (1.17–2.58)	0.007	1.79 (1.23–2.60)	0.003
Nodal stage (N+ vs. N0)	2.58 (1.70–3.91)	<0.001	2.37 (1.60–3.51)	<0.001
HPV (positive vs. negative)	0.35 (0.16–0.74)	0.006		
Number of CD3+ cells (>150 vs. 0–149)	0.39 (0.21–0.73)	0.003		

*HPV* Human papillomavirus, *OPSCC* oropharyngeal squamous cell carcinoma, *CI* confidence interval

In HPV-negative patients, tumor size and nodal stage were correlated with OS in univariate analysis. Neither number of infiltration of T-cells nor SERPINB1 expression was of significant importance. In HPV-positive tumors, a high number of intratumoral CD3+ cells, low expression of SERPINB1, and younger age were associated with a significant better OS (Table 3; Fig. 2). As the group of HPV-positive patients was rather small and only 10 deaths occurred in this subgroup, multivariate analysis was not possible. In HPV-negative patients, multivariate analysis showed that a high T- or N-stage were independently associated with a worse OS, while CD3+ cell influx was not (Table 4).

## Discussion

Previous studies showed that the immune system is involved in the process of carcinogenesis of HNSCC and elevated levels of tumor-infiltrating lymphocytes (TILs) are associated with an improved survival [9, 25]. Considering the poor survival rates, HNSCC patients may therefore benefit from additional immunotherapy in the future. In this study, we evaluated the amount of CD3+, CD4+, CD8+ TILs, and expression of granzyme inhibitors SERPINB1, SERPINB4, and SERPINB9 in relation to HPV-status and survival in OPSCC. In our study, HPV-positive tumors showed to have a significantly higher number of CD3+, CD4+, and CD8+ TILs. A high number of CD3+ TILs correlated with improved survival, independent of HPV status. In addition, we show for the first time that OPSCCs express granzyme inhibitors (SERPINB1, SERPINB4, and SERPINB9) to evade granzyme-induced cytotoxicity. Their expression was not different between HPV-positive and HPV-negative tumors. However, a high SERPINB1 expression identified a subgroup of HPV-positive tumors with a poor survival, which is comparable to HPV-negative tumors.

In OPSCC, HPV status is a prognostic biomarker as HPV-positive tumors have a better long-term survival compared to HPV-negative OPSCC [1]. Moreover, HPV-positive OPSCC represents a distinct entity with different tumor biology reflected on genetic and epigenetic levels [26, 27]. We hypothesized that the immune response and expression of granzyme inhibitors may also differ between HPV-positive and HPV-negative tumors. In our study, the immune profiles of HPV-positive and HPV-negative OPSCC were distinct, notable for differences in levels of CD3+, CD8+, and CD4+ TILs. This suggests that viral proteins released by HPV-positive tumors might be presented in MHC class I and II molecules on the surface and elicit a strong immune response after recognition by CD4+ and CD8+ T-cells. This pro-immunogenic effect of HPV triggers an anti-tumorigenic immune response, which might partly explain the improved survival in this group. These findings are in line with the previous studies, although not in all studies the differences were statistically significant [9–11, 28–34]. This could be explained by the variation in HPV detection methods, difference in used antibodies for assessing of intratumoral lymphocytes and used cutoff values.

As HPV-positive patients have an improved survival compared to those with HPV-negative OPSCC, it could be that less intensive treatment regimens in HPV-positive tumors could achieve similar efficacy with less side effects. Therefore, several clinical trials are currently investigating different ways to de-escalate treatment and still receive local control [35]. However, several studies showed that not all HPV-positive OPSCC have an excellent survival [4, 36]. Gillison et al. [36] showed that heavy smoking patients with a HPV-positive tumor have reduced survival, almost comparable to patients with HPV-negative tumors. Additionally, Ward et al. [9] developed a prognostic model suggesting that patients with HPV-positive tumors with low levels of tumor-infiltrating lymphocytes have similar survival rates to HPV-negatives. This is in line with the findings in our cohort showing that elevated levels of CD3+ cells are an independent predictor for improved survival of OPSCC comparable in strength to HPV status as prognosticator. A stratified analysis in HPV-positive and HPV-negative patients showed that an increased influx of CD3+ cells in HPV-positive patients is significant associated with an improved survival. In contrast, in HPV-negative patients, this association reached no significance. These results suggest that the immune system plays a more important role in the clinical behavior of HPV-positive OPSCC than in HPV-negative OPSCC.

SERPIN family members play pivotal roles in the inhibition of serine proteases in blood coagulation, mental diseases, and innate immune responses [37]. Studies with knock out of SERPINB1 in mice showed a significant role for SERPINB1 in protecting lung antimicrobial proteins from proteolysis during microbe infection and its regulatory role in sustaining the balance of neutrophil reserve [38, 39]. Increasing evidence shows that granzyme H participates in the defense of inflammation and SERPINB1 is identified as a possible inhibitor [19]. This study identified that a high expression of SERPINB1 is associated with a worse prognosis in HPV-positive patients and could be a mechanism of the tumor to escape from recognition or killing by the immune system. A possible explanation is that the immunogenic response is higher in HPV-positive OPSCC, and therefore, the effect of granzyme inhibition is more effective in HPV-positive tumors. Alternatively, Tseng et al. [40] showed that SERPINB1 might have a role in promoting motility in oral cancer cells and increasing invasiveness. This indicates a correlation with tumor metastases and therefore a worse prognosis.

In selecting patients properly for immunotherapy, it is important to account for the presence of suppressive mechanisms of immune system. The granule exocytosis pathway is the major mechanism via which cytotoxic lymphocytes, including natural killer (NK) cells and cytotoxic T-lymphocytes, counteract virus-infected cells and tumor tissue. The principal mechanism of killing in this pathway is by the release of granules containing the pore-forming protein perforin and a family of structurally homologues serine proteases known as granzymes into the immunological synapse. Pore formation induced by perforin facilitates the introduction of granzymes into the cell. In the cell, they can induce apoptosis by the cleavage of intracellular substrates [14, 41]. Humans express five different granzymes: granzyme A, B, H, K, and M [16]. All these granzymes can induce cell death, although each granzyme induces a unique pathway that only overlaps partially. The balance between activation and inhibition of the proteolytic cascade must be tightly controlled to avoid self-damage by, for example, intracellular leakage of granzymes. Additionally, tumor and virus-infected cells use strategies to evade granzyme induced cell death. It has been well established that granzyme B activity is controlled by SERPINB9 [42]. Recently, two other granzyme inhibitors were identified. SERPINB1 has been identified as a granzyme H inhibitor and SERPINB4 as a granzyme M inhibitor [17, 19]. Therefore, we assessed the expression of these three granzyme inhibitors. Multiple studies show that expression of SERPINB9 in melanomas is correlated with worse survival and poor response to immunotherapy [43]. For HNSCC, the expression of granzyme inhibitors has not been studied yet. In this study, we show for the first time that SERPINB9, SERPINB1, and SERPINB4 are not expressed in normal tissue, but expression can be acquired in OPSCC. There was no difference in expression of the studied SERPINS between HPV-positive and HPV-negative tumors. No correlation was found between the expression of SERPINS and the number of TILs. The observation of expression of granzyme inhibitors in OPSCC is important as it may affect the success rate of immunotherapy in OPSCC.

The results of study should be viewed within the constraints of several limitations. First, TMA’s were used for the evaluation of the IHC stainings. Although TMA is accepted nowadays as a fast and accurate approach for the evaluation of IHC stainings in large groups, it must be denoted that it could introduce possible bias due to the high heterogeneity of HNSCC. Second, overexpression of SERPINB1 in HPV-positive OPSCC showed a significant association with worse survival, but the low number of patients and events (*N* = 10) in the HPV-positive group did not allow for multivariate analysis. Therefore, our results should be validated in a cohort with higher numbers of HPV-positive patients, and analyses should be stratified for therapy.

In conclusion, it is plausible that the immune system plays a more important role in carcinogenesis of virally induced oropharynx tumors. Consequently, HPV-positive patients with a high expression of SERPINB1 or low invasion of CD3+ cells should receive additional therapy instead of treatment de-escalation.

## List of abbreviations

AJCC: American Joint Committee on Cancer
CCRT: Cisplatinum-based chemoradiotherapy
CI: Confidence interval
FFPE: Formalin-fixed paraffin-embedded
Gr: Granzymes
HNSCC: Head and neck squamous cell carcinoma
HPV: Human papillomavirus
IHC: Immunohistochemistry
OPSCC: Oropharyngeal squamous cell carcinoma
OS: Overall survival
PORT: Postoperative radiotherapy
ROC: Receiver operating characteristic
RT: Radiotherapy
TILs: Tumor-infiltrating lymphocytes
TMA: Tissue microarray
